# First person – Xinrui Wang

**DOI:** 10.1242/dmm.047829

**Published:** 2020-12-21

## Abstract

First Person is a series of interviews with the first authors of a selection of papers published in Disease Models & Mechanisms, helping early-career researchers promote themselves alongside their papers. Xinrui Wang is first author on ‘*Myh6*-driven Cre recombinase activates the DNA damage response and the cell cycle in the myocardium in the absence of loxP sites’, published in DMM. Xinrui is a postdoctoral fellow in the lab of John Auchampach at the Medical College of Wisconsin, Milwaukee, WI, USA, investigating cellular and molecular mechanisms of myocardium re-muscularization after injury.


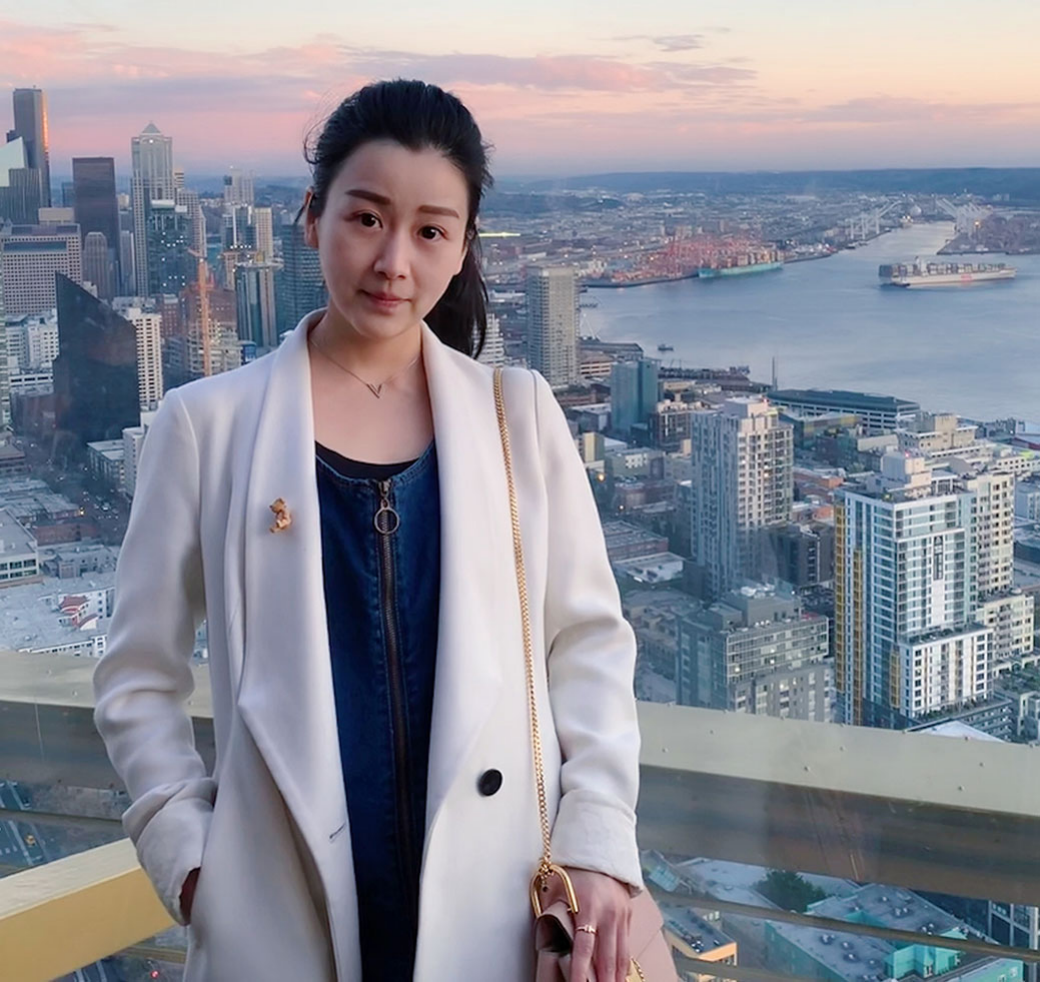


**Xinrui Wang**

**How would you explain the main findings of your paper to non-scientific family and friends?**

Every year nearly 1 million Americans experience a myocardial infarction, or what is commonly called a ‘heart attack’. A myocardial infarction occurs when a major blood vessel becomes clogged, thereby starving the heart of oxygen. If the clot is not cleared quickly to restore blood flow, the heart muscle dies and becomes permanently damaged. Because of this, it may not be able to pump blood as much as is required for the person to remain healthy or even to stay alive. A major objective of cardiovascular research is to identify potential therapeutic targets that will allow heart muscle to regrow after it has been damaged by a heart attack. For this purpose, cardiovascular researchers commonly utilize a ‘tool’ called the Cre-loxP system, which is designed to remove a specific gene in heart muscle cells of experimental animals including mice. While this is a very useful tool to determine the importance of a potential therapeutic target during heart repair, it is known to produce non-specific side effects, some of which I have identified in my research. It is very important for researchers that use this model system to be aware of these off-target effects, so that appropriate controls are included in their experiments and to prevent erroneous interpretation of results. Better understanding of genetic tools like the Cre-loxP system will aid in the discovery of therapies to treat people who have suffered a heart attack.

**What are the potential implications of these results for your field of research?**

During a recent study utilizing the tamoxifen-activated *Myh6-merCremer* transgene to recombine a loxP-flanked target, we observed that loxP-free control mice expressing *merCremer* exhibited cardiac defects. Of importance, with regard to cardiac regeneration studies, we observed evidence that the presence of merCremer in the nucleus induces DNA damage and unscheduled cell-cycle activation in cardiomyocytes as well as in non-cardiomyocytes. Moreover, activation of merCremer exacerbates dysfunction and apoptosis following experimental myocardial infarction. These findings indicate that studies employing constitutively active Cre recombinase, or tamoxifen-induced *merCremer*, to recombine loxP-flanked target genes, should anticipate the occurrence of off-target effects and be appropriately controlled.

“[…] however useful the Cre-loxP system may be, it is not without flaws.”

**What are the main advantages and drawbacks of the model system you have used as it relates to the disease you are investigating?**

During the past two decades, experiments utilizing constitutive as well as tamoxifen-activated *Myh6*-driven *Cre* transgenes have led to remarkable discovery and insight into the roles of many genes involved in all aspects of cardiology. It is of concern that, despite the potential for the side effects of Cre to obfuscate experimental results when using Cre-loxP genetic models, many published reports do not adequately control for the possible effects of Cre alone (loxP free). The inclusion of appropriate controls, coupled with an awareness of potential defects that may be mediated by Cre alone, are required to avoid misinterpreting results using Cre-loxP models. My findings will hopefully emphasize the fact that however useful the Cre-loxP system may be, it is not without flaws. The effects of Cre recombinase alone may be significant and may even lead to unpredicted positive research outcomes.

**What has surprised you the most while conducting your research?**

Many of my observations were unanticipated. It surprised me that Myh6-merCremer activation exacerbated the extent of cardiac dysfunction caused by myocardial infarction, which is an important observation since *Myh6-merCremer* transgenic mice are widely used in cardiac regeneration studies to assess the effects of altering the expression of loxP-targeted genes after cardiac injury. I was also surprised by our observation that the presence of Myh6-merCremer in the nucleus of cardiomyocytes caused unscheduled cell-cycle activation in the complete absence of loxP sites. This occurred not only in cardiomyocytes, but also in other unidentified cell types found in the myocardium. Notably, Cre activation induced the expression of several different cell-cycle genes to levels that were extraordinarily high. Effects on non-cardiomyocytes likely reflect a paracrine-induced inflammatory response or perhaps compensatory changes in response to the cardiac dysfunction.

**Figure:**
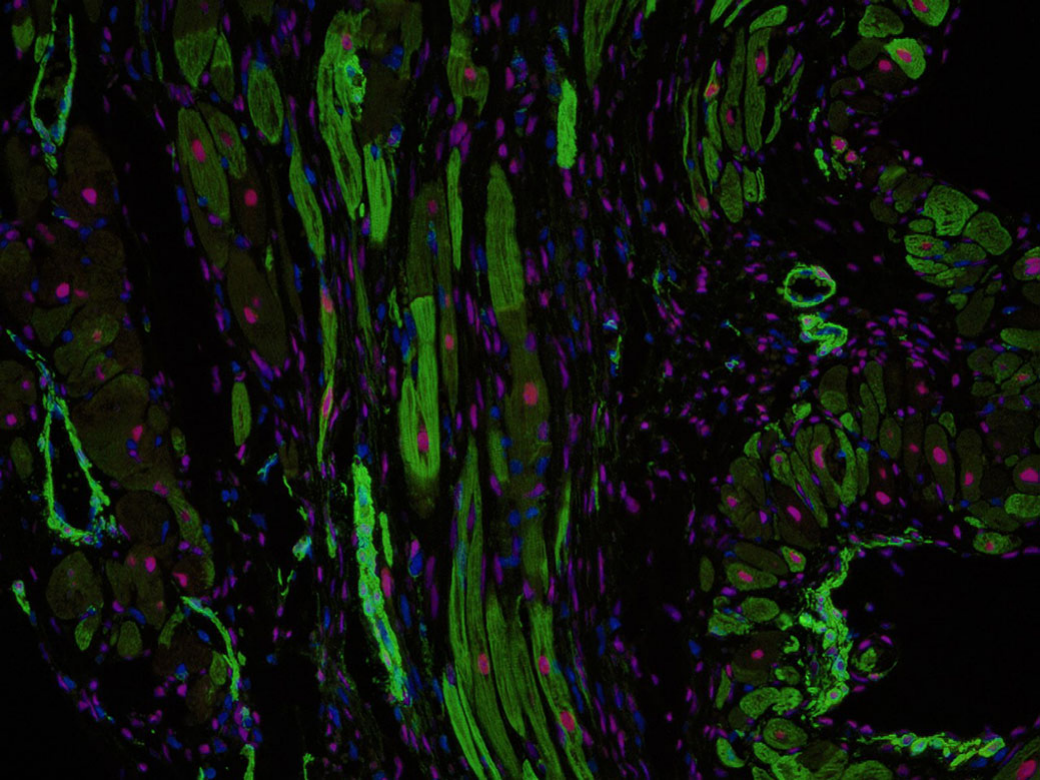
**α-Smooth muscle actin (green) and GATA4 (red) immunostaining of an infarcted mouse heart.**

**Describe what you think is the most significant challenge impacting your research at this time and how will this be addressed over the next 10 years?**

After many years of unsuccessful exogenous means to trigger heart regeneration, attention has turned to the heart's intrinsic mechanisms of re-muscularization. Research to understand endogenous cardiac regeneration is abundant but still limited. It is important to continue to decipher the cellular and molecular mechanisms of modulation of the regeneration capacity in the adult mammalian heart. Because the non-proliferative status of adult cardiomyocytes may be an important evolutionary adaptation to optimize cardiac function, research must continue to understand how to best manipulate the regulatory mechanisms just enough to permit regeneration without functional consequences. And because analysis of cell division in the mammalian heart is complicated by cardiomyocyte binucleation, this makes it challenging to interpret traditional assays of cell turnover. Markers of cardiomyocyte proliferation (rather than cell-cycle activity) and/or appropriate animal models will need to be identified or developed to address this major challenge in the field.

“Getting individual funding encourages and empowers trainees to continue to pursue their career goals.”

**What changes do you think could improve the professional lives of early-career scientists?**

More fellowship and funding opportunities would definitely help to improve the professional lives of early-career scientists. Getting individual funding encourages and empowers trainees to continue to pursue their career goals.

**What's next for you?**

The objective of my postdoctoral training is to define the role of the acetyltransferase Tip60 in the myocardium. I'm testing the hypothesis that Tip60 critically functions in cardiomyocytes to maintain proliferative senescence and to inhibit apoptosis, and that its depletion or temporary inactivation is both pro-regenerative and cardioprotective following ischemia. The broad goal of my research is to identify molecular pathways that can be targeted to facilitate heart regeneration and to develop therapies to aid people that have experienced a myocardial infarction. I look forward to becoming an independent investigator and to becoming a productive contributor to the scientific community.
